# Cause-specific mortality among patients with cirrhosis in a population-based cohort study in Ontario (2000–2017)

**DOI:** 10.1097/HC9.0000000000000194

**Published:** 2023-06-28

**Authors:** Peter L. Wang, Maya Djerboua, Jennifer A. Flemming

**Affiliations:** 1 Departments of Medicine, Kingston, Ontario, Canada; 2 ICES, Queen’s University, Kingston, Ontario, Canada; 3 Public Health Sciences, Queen’s University, Kingston, Ontario, Canada

## Abstract

Although patients with cirrhosis are at increased risk of death, the exact causes of death have not been reported in the contemporary era. This study aimed to describe cause-specific mortality in patients with cirrhosis in the general population.

**Methods:**

Retrospective cohort study using administrative health care data from Ontario, Canada. Adult patients with cirrhosis from 2000–2017 were identified. Cirrhosis etiologies were defined as HCV, HBV, alcohol-associated liver disease (ALD), NAFLD, or autoimmune liver disease/other with validated algorithms. Patients were followed until death, liver transplant, or end of study. Primary outcome was the cause of death as liver-related, cardiovascular disease, non-hepatic malignancy, and external causes (accident/self-harm/suicide/homicide). Nonparametric analyses were used to describe the cumulative incidence of cause-specific death by cirrhosis etiology, sex, and compensation status.

**Results:**

Overall, 202,022 patients with cirrhosis were identified (60% male, median age 56 y (IQR 46–67), 52% NAFLD, 26% alcohol-associated liver disease, 11% HCV). After a median follow-up of 5 years (IQR 2–12), 81,428 patients died, and 3024 (2%) received liver transplant . Patients with compensated cirrhosis mostly died from non-hepatic malignancies and cardiovascular disease (30% and 27%, respectively, in NAFLD). The 10-year cumulative incidence of liver-related deaths was the highest among those with viral hepatitis (11%–18%) and alcohol-associated liver disease (25%), those with decompensation (37%) and/or HCC (50%–53%). Liver transplant occurred at low rates (< 5%), and in men more than women.

**Conclusions:**

Cardiovascular disease and cancer-related mortality exceed liver-related mortality in patients with compensated cirrhosis.

## Graphical abstract

**Figure:**
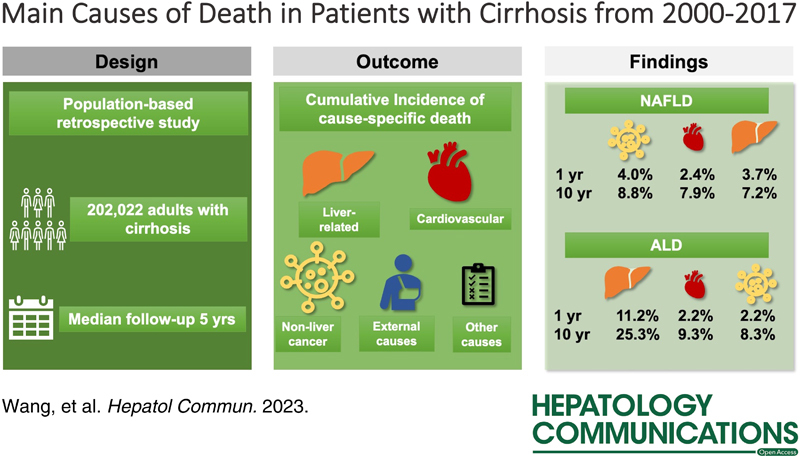

## INTRODUCTION

Mortality in patients with cirrhosis is high, estimated to be 5 times that of the general population, and the presence of hepatic decompensation further doubles this risk.^1^ Recent studies have also shown that deaths from cirrhosis are rising in North America and globally.^2^ This increase in mortality is expected to be further compounded by the projected doubling of incident cirrhosis by 2040, primarily from alcohol-associated liver disease (ALD) and NAFLD.^3^

Although it is recognized that deaths secondary to liver disease are rising, the specific causes of death among patients living with cirrhosis have not been well described. Furthermore, although etiologies of chronic liver disease have been linked with different extrahepatic conditions (eg, NAFLD with metabolic syndrome), whether that translates to an increase in death due to those extrahepatic conditions is not known at the population level. That is explained by the fact that studies typically evaluate national death registries which are unable to define non–liver-related causes of death among those with cirrhosis.^4–8^ Epidemiological studies describing cause-specific mortality in individuals with cirrhosis are scarce and typically from historic cohorts before the year 2000, and all have been outside of North America.^9–11^ Therefore, there is a lack of understanding of the causes of death among patients with cirrhosis in a contemporary era with advances in treatment (eg, direct-acting antivirals) or shifting etiologies of cirrhosis.

Understanding the cause of death among patients with cirrhosis at a population level is important as it may inform preventative public health efforts as well as individual chronic disease management. The aim of our study was to use population-level data to describe the cumulative incidence of cause-specific mortality in patients with cirrhosis, stratified by the main etiologies of cirrhosis and decompensation status.

## METHODS

### Study design, databases, and cohort identification

This was a population-based retrospective cohort study in Ontario, Canada. This study used routinely collected administrative health care data held at ICES (formerly the Institute for Clinical Evaluative Sciences). ICES is a nonprofit, independent research institute whose status under Ontario’s health information privacy law allows it to legally store and analyze individual-level personal health information for the purposes of health system evaluation and improvement. Ontario is Canada’s most populous province of over 14.9 million individuals comprising 40% of the population of Canada and 30% of whom self-identify as a visible minority. The estimated annual provincial out-migration leading to loss to follow-up is less than 0.5%. All residents of Ontario have universal access to health coverage through the Ontario Health Insurance Plan (OHIP), a single payer for all medically necessary services. The primary databases used in this study included (a) the Canadian Institute of Health Information Discharge Abstract Database and the National Ambulatory Care Reporting System, which capture all inpatient, ambulatory and emergency room services; (b) the Ontario Laboratories Information System, which contains over 90% of the entire provincial laboratory data; (c) the Registered Persons Database and the Ontario Registrar General database, which contains demographic and vital statistics including death data, and the cause of death to the end of 2017; (d) the Canadian Organ Replacement Registry, which is a national database of organ transplantation of patients with end-stage organ failure, Public Health Ontario Laboratories, which includes viral hepatitis results for >95% of the province; (e) Ontario Health Insurance Plan (OHIP), which captures all billable inpatient and outpatient services provided by physicians; (f) Ontario Cancer Registry, which contains information on the incidence, mortality and survival of cancer in Ontario; and (g) Postal Code Conversion File, which includes postal codes for Ontario residents. These data sets were linked using unique encoded identifiers and analyzed at ICES. This study was approved by the Queen’s University Health Sciences and Affiliated Teaching Hospitals Research Ethics Board (DMED 1651-13). No patient written informed consent was required, and the need for patient written informed consent was waived by the Queen’s University Health Sciences Research Ethics Board. The study protocol conformed to the ethical guidelines of the 2013 Declaration of Helsinki protocol, and abide by the 2018 Declaration of Istanbul. This study adhered to the Strengthening the Reporting of Observational Studies in Epidemiology^12^ and Reporting of studies Conducted using Observational Routinely collected health Data guidelines.^13^ All adults over 18 years old from January 1, 2000 to December 31, 2017, with prevalent or incident cirrhosis were identified using previously validated administrative coding, with associated sensitivity of 99% and specificity of 79% and a 5-year lookback window.^14^ Patients were excluded if they lacked a unique ICES identifier or had less than 1 year of OHIP eligibility.

### Patient demographics and covariates

Baseline patient characteristics were defined at the time of cohort entry. Baseline demographics included age, sex, and income quintile defined based on residential postal code linked to Statistics Canada census data (https://www150.statcan.gc.ca/n1/pub/82-221-x/2013001/quality-qualite/qua8-eng.htm). Patients were assigned as living in a rural area based on postal codes taken from Statistic’s Canada Postal Code Conversion File where the community size is less than 10,000 people within a census metropolitan area or census agglomeration. The most likely etiology of cirrhosis was determined as viral hepatitis C (HCV), viral hepatitis B (HBV), alcohol-associated liver disease (ALD), NAFLD, or autoimmune liver disease (AI)/other using a validated hierarchical algorithm, which includes viral serology (positive HBV surface antigen, HBV DNA, HCV RNA), and International Classification of Diseases and OHIP billing codes from the Public Health Ontario Laboratories, Ontario Laboratories Information System, Discharge Abstract Database, OHIP and National Ambulatory Care Reporting System databases (sensitivity of >75% and specificity of >90% for all causes).^15^ Due to its hierarchical nature as each criterion is applied, this algorithm does not permit the identification of 2 or more concurrent chronic liver diseases (eg, a patient with HCV cirrhosis cannot be labeled as having NAFLD as well). Patients were considered to have decompensation (ascites, variceal bleed, HE, hepatorenal syndrome) and/or HCC at or any point during follow-up from cohort entry by using validated case definitions.^16^ Model for end-stage liver disease-sodium score was calculated using the individual values of total bilirubin, serum creatinine, sodium and international normalized ratio from the Ontario Laboratories Information System data.^17^ The closest lab values within 1 year of cohort entry were used to calculate the model for end-stage liver disease-sodium score. Comorbidities were defined using validated databases for hypertension,^18^ diabetes,^19^ congestive heart failure,^20^ HIV,^21^ and administrative coding for the history of substance misuse, and using the Charlson Comorbidity Index (CCI) using a 2-year lookback. The number of patients who had a non-hepatic malignancy diagnosed either before or subsequent to cohort entry was identified from the Ontario Cancer Registry.

### Outcomes

The primary outcome was the cause of death determined using the International Classification of Diseases coding from the Ontario Registrar General defined based on the top causes of deaths by Statistics Canada and categorized as either: (a) liver-related as previously defined,^22^ which includes also includes primary liver malignancies (HCC and intrahepatic cholangiocarcinoma); (b) cardiovascular disease (CVD: cardiovascular, cerebrovascular, and diabetes); (c) non-hepatic malignancy (all cancers except primary liver); (d) accident/self-harm/suicide/homicide or; (e) Other (all other causes of death). The outcome of liver transplant (LT) was defined using administrative coding as outlined in the Supplement. A full list of codes used are available in Supplemental Table 1, http://links.lww.com/HC9/A341. The frequency and proportion of deaths by subtypes of non-hepatic malignancies (colorectal, breast, lung, prostate, and other) are provided in Supplemental Table 2, http://links.lww.com/HC9/A341. The number of patients with malignancy diagnosed before or during follow-up is shown in Supplemental Table 3, http://links.lww.com/HC9/A341.

### Statistical approach

Patients were followed from cohort entry until death, liver transplant, or December 31, 2017, whichever came first. Descriptive statistics were calculated for the entire cohort and for each cirrhosis etiology. Categorical data were summarized by frequency with percentages. Continuous data were summarized by median and interquartile range. Because of an ICES privacy agreement, data containing small cells (n < 6) are not reportable due to re-identification risk. The proportion of deaths by each death category outlined above was stratified by cirrhosis etiology, compensation status, and the presence of HCC. The cumulative incidence of cause-specific mortality was defined using nonparametric analyses of each cause-specific mortality with other causes of mortality and liver transplant as competing risks. This was stratified by etiology of cirrhosis, compensation status, and sex. The cumulative incidence of cause-specific mortality stratified by first decompensating events are reported in Supplemental Table 4, http://links.lww.com/HC9/A341. The level of statistical significance was *p* < 0.05. All data were analyzed using SAS version 9.4 (SAS Institute Inc., Cary, North Carolina, USA).

## RESULTS

### Cohort demographics

We identified n = 202,022 patients with an incident or prevalent cirrhosis who were followed for a median of 5 years (IQR 2–12 y, Table 1, Supplemental Fig. 1, http://links.lww.com/HC9/A341). The median age at cohort entry was 56 years (IQR 46–67 y), 60% were male, 73% were compensated, 22% had decompensated disease, 3% had HCC, and 3% had both decompensation and HCC. Most patients had NAFLD as the cause of cirrhosis (52%), 26% had ALD, and 11% had HCV. The majority lived in urban areas (88%) and in neighborhoods of lower-income quintiles. During follow-up, n = 81,428 (40%) died, and only n = 3,024 (2%) received a liver transplant, with the highest proportion receiving liver transplant among patients with AI/other (7%) while LT was rare in those with ALD (1%) and NAFLD ( < 1%) (Table 1).

**TABLE 1:** Baseline demographics and clinical features of patients by etiology of cirrhosis.

	All	NAFLD	ALD	HCV	AI/other	HBV	*p*
	(n = 202,022)	(n = 104,736)	(n = 53,060)	(n = 22,865)	(n = 11,399)	(n = 9,962)	—
Age, y, md (IQR)	56 (46–67)	58 (45–70)	57 (48–66)	52 (44–59)	57 (46–68)	50 (40–60)	< 0.001
Sex, female, n (%)	81,401 (40.3)	50,493 (48.2)	14,166 (26.7)	7,289 (31.9)	6157 (54)	3296 (33.1)	< 0.001
Rurality, urban, n (%)	176,809 (87.5)	92,398 (88.2)	44,481 (83.8)	20,552 (89.9)	9623 (84)	9755 (97.9)	< 0.001
Income quintile, n (%)	—	—	—	—	—	—	< 0.001
** **1	48,190 (23.9)	22,273 (21.3)	13,950 (26.3)	7223 (31.6)	2294 (20.1)	2450 (24.6)	—
** **2	42,656 (21.1)	21,677 (20.7)	11,332 (21.4)	5038 (22.0)	2308 (20.2)	2301 (23.1)	—
** **3	38,707 (19.2)	20,750 (19.8)	9613 (18.1)	4167 (18.2)	2195 (19.3)	1982 (19.9)	—
** **4	36,297 (18.0)	20,240 (19.3)	8643 (16.3)	3474 (15.2)	2148 (18.8)	1792 (18.0)	—
5	33,087 (16.4)	18,832 (18.0)	7958 (15.0)	2722 (11.9)	2197 (19.3)	1378 (13.8)	—
** **Missing	3085 (1.5)	964 (0.9)	1564 (2.9)	241 (1.1)	257 (2.3)	59 (0.6)	—
CCI, n (%)	—	—	—	—	—	—	< 0.001
** **0	164,039 (81.2)	86,331 (82.4)	40,285 (75.9)	19,805 (86.6)	8622 (75.6)	8996 (90.3)	—
** **1	13,628 (6.7)	5597 (5.3)	5390 (10.2)	1357 (5.9)	1009 (8.9)	275 (2.8)	—
** **2-3	15,305 (7.6)	7634 (7.3)	4962 (9.4)	1131 (4.9)	1135 (10.0)	443 (4.4)	—
** **4+	9050 (4.5)	5174 (4.9)	2423 (4.6)	572 (2.5)	633 (5.6)	248 (2.5)	—
Decompensation or HCC, n (%)	—	—	—	—	—	—	< 0.001
** **Compensated	146,645 (72.6)	87,896 (83.9)	30,218 (57.0)	14,616 (63.9)	6601 (57.9)	7314 (73.4)	—
** **Decompensation only	43,801 (21.7)	13,384 (12.8)	20,336 (38.3)	5171 (22.6)	3561 (31.2)	1349 (13.5)	—
** **HCC only	4983 (2.5)	1931 (1.8)	782 (1.5)	1086 (4.7)	503 (4.4)	681 (6.8)	—
** **Both decompensation and HCC	6593 (3.3)	1525 (1.5)	1724 (3.2)	1992 (8.7)	734 (6.4)	618 (6.2)	—
Decompensation type ─n (%)	—	—	—	—	—	—	< 0.001
** **HE	12,309 (24.4)	3979 (26.7)	5066 (22.9)	1675 (23.4)	1183 (27.5)	406 (20.6)	—
** **Hepatorenal syndrome	1,575 (3.1)	323 (2.2)	1,027 (4.7)	115 (1.6)	84 (1.9)	26 (1.3)	—
** **Variceal bleeding	14,578 (28.9)	3732 (25.0)	5254 (23.8)	3145 (43.9)	1515 (35.3)	932 (47.3)	—
** **Jaundice	1,598 (3.2)	556 (3.7)	683 (3.10	85 (1.2)	239 (5.5)	35 (1.8)	—
** **Ascites	20,334 (40.4)	6319 (42.4)	10,030 (45.5)	2143 (29.9)	1274 (29.7)	568 (28.9)	—
Congestive heart failure, n (%)	20,446 (10.1)	12,499 (11.9)	5916 (11.1)	793 (3.5)	982 (8.6)	256 (2.6)	< 0.001
Hypertension, n (%)	90,922 (45.0)	50,877 (48.6)	24,989 (47.1)	7136 (31.2)	5096 (44)	2824 (28.3)	< 0.001
Diabetes mellitus, n (%)	70,667 (35.0)	38,960 (37.2)	18,182 (34.3)	6768 (29.6)	4256 (37)	2501 (25.1)	< 0.001
Substance misuse, n (%)	13,186 (6.5)	2354 (2.3)	5852 (11.0)	4446 (19.4)	351 (3.1)	183 (1.8)	< 0.001
HIV, n (%)	1340 (0.7)	389 (0.4)	160 (0.30)	549 (2.4)	33 (0.3)	209 (2.1)	< 0.001
MELD-Na score, md (IQR)	9 (7–15)	9 (6–14)	13 (9–19)	8 (6–11)	11 (8–17)	7 (6–10)	< 0.001
** **Available, n (%)	54,098 (26.8)	26,360 (25.2)	13,098 (24.7)	7967 (34.8)	2643 (23.2)	4030 (40.5)	< 0.001
Follow-up, years, md (IQR)	5 (2–12)	6 (2–12)	4 (2–10)	6 (3–12)	7 (3–13)	7 (3–12)	< 0.001
Liver transplant by the end of follow-up, n (%)	3,204 (1.6)	565 (0.5)	565 (1.1)	1,016 (4.4)	750 (6.6)	308 (3.1)	< 0.001
Total deaths by end of follow-up, n (%)	81,428 (40.3)	34,413 (32.9)	32,810 (61.8)	7048 (30.8)	5427 (47.6)	1730 (17.4)	< 0.001

Abbreviations: ALD, alcohol-associated liver disease; CCI, Charlson Comorbidity Index; CVD, cardiovascular disease; MELD-Na, model for end-stage liver disease.

### Causes of death overall and stratified by cirrhosis etiology

**FIGURE 1:**
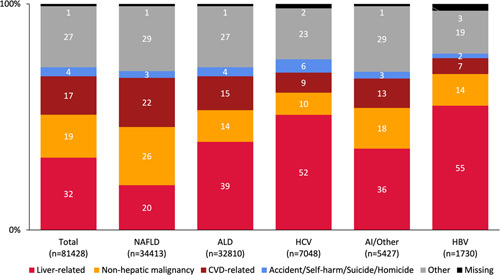
Proportion of deaths (n = total number of deaths) for each etiology of cirrhosis by cause of death. Abbreviations: AI, Autoimmune liver disease; ALD, alcohol-associated liver disease; CVD, cardiovascular disease.

Causes of death stratified by cirrhosis etiology are shown in Fig. 1. During the study period, the most common cause of death was liver-related (32%), followed by non-hepatic malignancy (19%) and CVD-related causes (17%). When stratified by cirrhosis etiology, the proportion of liver-related deaths was highest among those with viral hepatitis (HBV 56%; HCV 52%), followed by ALD (39%), and was lowest in those with NAFLD (20%). Among those with NAFLD cirrhosis, the leading cause of death was non-hepatic malignancy (26%), followed by CVD-related (22%). Among the non-hepatic malignancies across cirrhosis etiologies, there was a higher proportion of patients with lung cancer in patients with ALD (29%) compared to other etiologies (13-22%) (Supplemental Table 2, http://links.lww.com/HC9/A341). External causes of death accounted for 6% of deaths in those with HCV cirrhosis and 4% in ALD (Fig. 1).

### Causes of death stratified by compensation status and HCC

**FIGURE 2:**
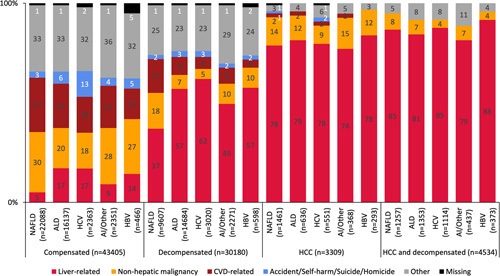
Proportion of deaths (n = total number of deaths) for each etiology of cirrhosis by cause of death and compensation status and presence of HCC. Cell sizes (excluding those in the missing category) smaller than 6 are suppressed per ICES policy to prevent the potential re-identification of individuals. Abbreviations: AI, Autoimmune liver disease; ALD, alcohol-associated liver disease; CVD, cardiovascular disease.

Causes of death stratified by decompensation status and etiology are shown in Figure 2. Across all etiologies, the minority of patients with compensated cirrhosis suffered a liver-related death (5-17%) and were most likely to die from non-hepatic malignancy (highest in compensated NAFLD: 30%) or a CVD-related death (highest in compensated NAFLD: 27%). Patients with compensated HCV had the highest proportion who suffered an external cause of death (13%), double that of all other etiologies of cirrhosis (2-6%). Conversely, among those with either decompensated cirrhosis and/or HCC, liver-related deaths were the most common cause of death (decompensated only: 37%–62%; HCC only: 74%–79%; HCC and decompensation: 79%–88%) (Fig. 2).

### Cumulative incidence of death and LT by cirrhosis etiology

**FIGURE 3:**
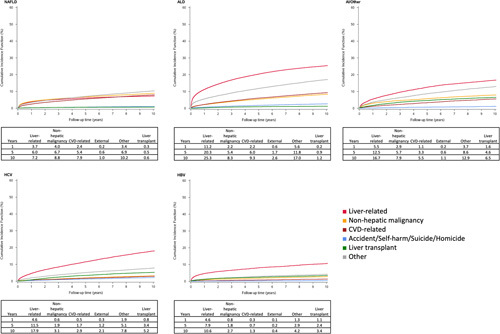
Cumulative incidence functions by etiologies of cirrhosis for the main causes of death and liver transplant as a competing risk. Abbreviations: AI, Autoimmune liver disease. ALD, alcohol-associated liver disease. CVD, cardiovascular disease.

The cumulative incidences over 10 years for the major causes of death by cirrhosis etiology are shown in Fig. 3. Overall, the 10-year cumulative incidence of liver-related mortality was highest in those with ALD (25%), followed by HCV (18%), and the lowest was among those with NAFLD (7%). In those with NAFLD cirrhosis, liver-related deaths were lower than those secondary to non-hepatic malignancy (9%) and were overtaken by CVD-related death after ~7 years of follow-up. Also, among the NAFLD group, it is also worth noting that “Other” causes of death combined exceed all other causes of mortality at 5 (7%) and 10 years (10%). Patients with ALD show a steep rise in liver-related mortality within the first year and a high 10-year cumulative incidence of deaths from liver-related causes (25%) and accident/self-harm/suicide/homicide (3%). For HCV, the cumulative incidence of liver-related deaths at 10 years is 18% and much higher compared to other causes. In those with viral hepatitis, there is a relatively higher 10-year cumulative incidence of liver transplant (3-5%), which is higher than non-hepatic malignancy (~3%) and CVD-related deaths (~1%–2%). There is also a relatively higher cumulative incidence of liver transplant in those with AI/other and viral hepatitis as causes of cirrhosis (3%–6% at 10 y), compared to those with ALD and NAFLD (0.6%–1.1% at 10 y).

### Cumulative incidence of death and LT by compensation status

**FIGURE 4:**
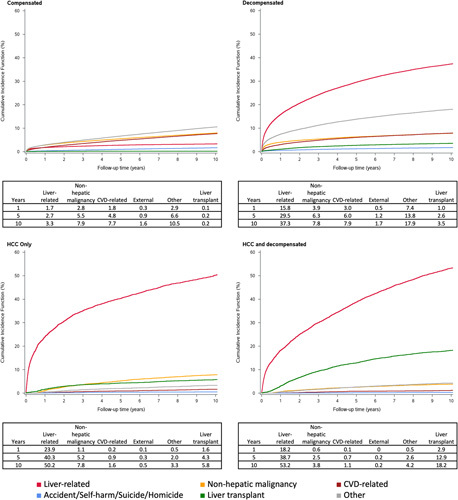
Cumulative incidence functions by compensation status and presence of HCC for the main causes of death and liver transplant as a competing risk. Abbreviation: CVD, cardiovascular disease.

Comparing the cumulative incidence by compensation status (Fig. 4), the 10-year cumulative incidence of non-hepatic malignancy (8%) and CVD-related deaths (8%) in compensated patients is higher than for liver-related deaths (3%), which plateaus over the 10-year period. The combined “Other” causes of death make up most of the deaths in compensated disease during the 10-year period (10%). In those with decompensated cirrhosis and those with HCC with and without decompensation, there is a steep rise in liver-related mortality in the first year (16%–24%) and greatly exceeds deaths from all other causes by 10 years (37%, 50% and 53% in those with decompensation, HCC and both, respectively). Compared to liver transplants at 10 years, there is also a relatively and progressively higher proportion of patients who receive liver transplants in those with decompensation (3%), HCC (6%), and those with both (18%).

### Cumulative incidence of death and LT by sex

**FIGURE 5:**
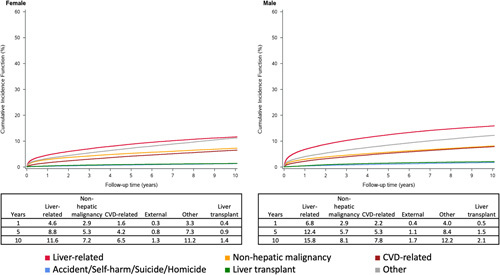
Cumulative incidence functions by sex for the main causes of death and liver transplant as a competing risk. Abbreviation: CVD, cardiovascular disease.

Over 10 years, female and male patients with cirrhosis had the same relative proportion of causes of death, in order from the highest to the lowest: liver-related (11%–16%), other causes (11%–12%), non-hepatic malignancy (7%–8%), CVD-related (6%–8%) and external causes (1%–2%) (Fig. 5). In women however, the cumulative incidence of other causes (11%) closely approaches that of liver-related deaths (12%), whereas this is not observed in men (12 vs. 16%, respectively). The cumulative incidence of liver transplant was lower for women compared to men at points over 10 years (10-year cumulative incidence 1% vs. 2%, respectively). Cumulative incidence functions of each etiology of liver disease stratified by sex are available in Supplemental Figures 2-6, http://links.lww.com/HC9/A341.

## DISCUSSION

In this retrospective population-based cohort of patients with cirrhosis over a median follow-up of 5 years, non-hepatic malignancy and cardiovascular disease-related deaths exceeded liver-related deaths among patients with compensated cirrhosis of any cause, with liver-related deaths being lowest in those with compensated NAFLD cirrhosis. Not surprisingly, in individuals with decompensated disease and/or HCC, liver-related causes of death become the most common cause of death, yet less than 5% received liver transplants during follow-up, with rates of transplant being lower among women compared to men. These data provide insight into the contemporary natural history of cirrhosis from a variety of etiologies at the level of the general population. Our results also identify that in addition to the management of underlying liver disease with a goal to prevent decompensation and HCC, the prevention and management of CVD and non-liver malignancy are key aspects when providing care for patients with compensated cirrhosis and will require multidisciplinary management. Finally, our results highlight that the majority of patients with decompensation and/or HCC do not receive liver transplants strengthening the importance of the need for goals of care discussions and the availability of services that can provide palliative care for this population.

To our knowledge, this is the first study to describe the causes of death in a population-based cohort of patients with cirrhosis from North America in a contemporary era with specific information on the etiology of cirrhosis, compensation status, and the presence of HCC. Historic studies from the UK and Denmark reported that liver-related causes of death occurred in ~half of the patients with cirrhosis.^9–11^ This is higher than our estimate of 32% of all deaths being liver-related. There may be several reasons for this, primarily related to differences between the cohorts. First, in our cohort, up to 16% had HBV or HCV-related cirrhosis compared to the other studies, which had only had ~10% from viral etiologies. Curative HCV treatments and improvements in HBV treatment and prevention may have resulted in lower liver-related mortality in our study era compared to prior studies. Two of the previous studies^9,10^ also included only patients with hospital admissions and, therefore, likely were enriched in those with decompensated disease and ALD cirrhosis (58%-61%), compared to our cohort, which also included compensated outpatients with just 26% having ALD cirrhosis.

Our study shows that in patients with ALD cirrhosis, there is much greater liver-related mortality, particularly in the first year after diagnosis. This extends a previous finding using death certificate data showing that in patients who died due to cirrhosis, the mortality burden is driven by alcohol use disorder and ALD.^2^ Adults under 40 years old are over-represented in a recent global study of people with harmful alcohol use.^23^ This underscores the importance of public health messaging and interventions, such as the recently updated publication on Canada’s alcohol consumption guideline that suggests that any amount of alcohol can be harmful.^24^ There is also a higher rate of lung cancer-related deaths in patients with ALD in our study. This likely reflects the higher rates of co-usage of nicotine with alcohol, and this should be a target of counseling for these patients. Although our study does find a relatively higher proportion of external causes of death in those with ALD cirrhosis, particularly those who are compensated, it does not specifically delineate which external cause of death, for example, suicide, homicide, accidents, or self-harm, is most prevalent. Alcohol use has been linked with violent deaths, including suicide and traumatic accidents.^25,26^ Support for addiction services and mental health are thus also critical for these patients to reduce overall mortality.

Metabolic disease and obesity are on the rise globally, and by extension, so too is the burden of NAFLD cirrhosis.^3^ Our results show that in patients with compensated cirrhosis, especially with NAFLD as the etiology, deaths due to CVD and non-hepatic malignancy exceeded liver-related mortality. Similar findings were noted in the UK study by Ratib et al where they found those with ‘unspecified cirrhosis,’ which likely represents undiagnosed NAFLD that had a higher risk of non–liver-related death, mostly due to non-hepatic neoplasms.^11^ Currently, general advice such as weight loss and alcohol abstinence, as well as the recent evidence for carvedilol,^27^ are the mainstays of preventing decompensation in NAFLD cirrhosis, although hepatoprotective and anti-fibrotic drugs may be in the pipeline. Therefore, the current optimal resource use would be to focus on interventions that improve cardiovascular health and age-appropriate malignancy screening in these patients at the primary care level.

The strengths of our study include the use of a large universally-insured, ethnically-diverse population, with a long 10-year follow-up for outcomes and previously validated definitions for cirrhosis and etiologies of liver disease. Furthermore, our analysis using a competing risk approach for cause-specific mortality is important for interpreting our results. Nonetheless, several key limitations in methodology bear mentioning. First, this study is retrospective and uses administrative codes, which may unavoidably have a misclassification bias. We try to mitigate this by using validated and accurate codes as described. Second, the use of the hierarchical algorithm for the etiologies of cirrhosis means that 2 etiologies may not be ascribed to 1 patient, which may be the case (eg, a patient with HCV and alcohol misuse would only be considered to have HCV). Third, the cause of death abstracted from death certificates may be inaccurate, and estimates on the accuracy of coding in the databases used are not available. Fourth, MELD scores were only available for ~25% of the patients based on lab values in the 1 year prior to cohort entry, which limits any interpretation. However, we intend this to be descriptive and are not using it as a predictor for any modeling. Finally, potentially important covariates, including race/ethnicity, BMI, prior antiviral use, and patterns of smoking and alcohol consumption, are not collected in ICES and thus cannot be considered.

## CONCLUSIONS

Those with cirrhosis due to NAFLD or compensated cirrhosis generally do not die of liver-related causes, and thus in addition to the prevention of further hepatic decline, efforts should also be directed at preventing and treating non-hepatic malignancy and cardiovascular comorbidities. Multidisciplinary and public health interventions to reduce ALD, eradicate viral hepatitis B and C, and prevent decompensation or the development of HCC will be paramount for preventing liver-related deaths.

## Supplementary Material

SUPPLEMENTARY MATERIAL
