# Supplementary material for: Cause-specific mortality among patients with cirrhosis in a population-based cohort study in Ontario (2000–2017)
Source: Hepatol Commun. 2023 Jun 28;7(7):e00194. doi: 10.1097/HC9.0000000000000194 (PMC10309525; doi:10.1097/HC9.0000000000000194)
Supplement: SUPPLEMENTARY MATERIAL [file hc9-7-e00194-s001.docx]

**Supplementary Tables**

Supplementary Table 1. List of codes for defining outcomes and covariates (including causes of death and liver transplantation).

| **Outcomes** | **Code Type** | **Codes** |
| --- | --- | --- |
| **Cause of Death** |  |  |
| Liver-related (includes liver diseases, hepatitis, and primary liver malignancies) | ICD-10 | K70, K71, K72, K73, K75, K76, I85, B15, B16, B17, B18, B19, C22 |
| Non-hepatic malignancies (includes all cancer except for primary liver malignancies) | ICD-10 | C0, C1, C2 (excluding C22), C3, C4, C5, C6, C7, C8, C91, C92, C93, C94, C95, C96, C97  Colorectal: C18, C19, C20  Lung: C33, C34  Breast: C50  Prostate: C61  Other: C0-C9, excluding C22, C18, C19, C20, C33, C34, C50, C61 |
| Cardiovascular disease-related (includes cardiovascular, cerebrovascular and diabetic causes) | ICD-10 | I00, I01, I02, I03, I04, I05, I06, I07, I08, I09, I11, I13, I2, I3, I4, I50, I51, I60, I61, I62, I63, I64, I65, I66, I67, I68, I69, E10, E11, E12, E13, E14 |
| Accident, self-harm, suicide or homicide | ICD-10 | V0, V1, V2, V3, V4, V5, V6, V7, V8, V9, W0, W1, W2, W3, W4, W5, W6, W7, W8, W9, X0, X1, X2, X3, X4, X5, X6, X7, X80, X81, X82, X83, X84, Y870, X85, X86, X87, X88, X89, X9, Y00, Y01, Y02, Y03, Y04, Y05, Y06, Y07, Y08, Y09, Y871 |
| **Liver Transplantation** |  |  |
| Living donor orthotopic liver transplantation | OHIP Fee | S266 |
| Deceased donor liver transplantation | OHIP Fee | S294 |
| **Covariates** |  |  |
| Hepatic Decompensation | ICD-9 | Hepatic failure/encephalopathy: 570, 5722, 5728  Variceal bleeding: 4560, 4562, 4568  Ascites: 7895  Hepatorenal syndrome: 5724  Jaundice: 7824 |
|  | ICD-10 | Hepatic failure/encephalopathy: K720, K721, K729  Variceal bleeding: I850, I864, I9820, I983  Ascites: R18  Hepatorenal syndrome: K767  Jaundice: R17 |
| Non-hepatic cancer diagnosis | ICD-O-3 Topography | Colorectal: C18, C19, C20  Lung: C33, C34  Breast: C50 Prostate: C61  Other: C0-C9, excluding C22, C18, C19, C20, C33, C34, C50, C61 |
| Substance misuse | ICD-9 | 2920, 2921, 2922, 2928, 2929, 3040, 3041, 3042, 3043, 3044, 3045, 3046, 3047, 3048, 3049, 3052, 3053, 3054, 3055, 3056, 3057, 3058, 3059 |
|  | ICD-10 | F11, F12, F13, F14, F15, F16, F18, F19 |
|  | OHIP ICD-9 | 292, 304 |

Supplementary Table 2. Frequency and proportion of deaths by each type of malignancy (N = number of deaths for each cirrhosis etiology). AI: Autoimmune liver disease. ALD: alcohol-associated liver disease. Cell sizes smaller than 6 are suppressed per ICES policy to prevent the potential re-identification of individuals. HBV: hepatitis B virus. HCV: hepatitis C virus. NAFLD: non-alcoholic fatty liver disease.

| **Cause and number of deaths** | **Total (N=81,428)** | **NAFLD (N=34,413)** | **ALD (N=32,810)** | **HCV (N=7,048)** | **AI/Other (n=5,427)** | **HBV (N=1,730)** |
| --- | --- | --- | --- | --- | --- | --- |
| **Non-hepatic malignancy, n (%)** | 15,174 | 8,784 | 4,495 | 679 | 982 | 234 |
| **Colorectal cancer** | 1,505 (9.9) | 970 (11.0) | 391 (8.7) | 50 (7.4) | 72 (7.3) | 22 (9.4) |
| **Breast cancer** | 607 (4.0) | 457 (5.2) | 95 (2.1) | 17 (2.5) | 31 (3.2) | 7 (3.0) |
| **Lung cancer** | 3,138 (20.7) | 1,527 (17.4) | 1,293 (28.8) | 147 (21.6) | 140 (14.3) | 31 (13.3) |
| **Prostate cancer** | 543 (3.6) | 332 (3.8) | 177 (3.9) | 15 (2.2) | 14 (1.4) | <6 (<2) |
| **Other non-hepatic cancer** | 9,381 (61.8) | 5,498 (62.6) | 2,539 (56.5) | 450 (66.3) | 725 (73.8) | 169 (72.2) |

Supplementary Table 3. Non-hepatic malignancy in the cohort identified at/prior to cohort entry and during follow-up.

|  | **All** | **NAFLD** | **ALD** | **HCV** | **AI/other** | **HBV** |
| --- | --- | --- | --- | --- | --- | --- |
| **Pre-existing non-hepatic cancer diagnosis – n (%)** |  |  |  |  |  |  |
| **Any non-hepatic malignancy** | 21,642 (10.7) | 13,558 (12.9) | 4,691 (8.8) | 1,252 (5.5) | 1,475 (12.9) | 666 (6.7) |
| **Colorectal** | 3,150 (14.6) | 2,050 (15.1) | 698 (14.9) | 113 (9.0) | 218 (14.8) | 71 (10.7) |
| **Breast** | 3,039 (14.0) | 2,173 (16.0) | 481 (10.3) | 133 (10.6) | 185 (12.5) | 67 (10.1) |
| **Lung** | 1,322 (6.1) | 777 (5.7) | 398 (8.5) | 70 (5.6) | 51 (3.5) | 26 (3.9) |
| **Prostate** | 2,984 (13.8) | 1,824 (13.5) | 843 (17.97) | 110 (8.8) | 155 (10.5) | 52 (7.8) |
| **Other non-hepatic malignancy** | 12,286 (56.8) | 7,551 (55.7) | 2,455 (52.3) | 867 (69.2) | 940 (63.7) | 473 (71.0) |
| **Non-hepatic cancer diagnosis during follow-up – n (%)** |  |  |  |  |  |  |
| **Any non-hepatic malignancy** | 22,529 (11.1) | 11,591 (11.1) | 6,912 (13.0) | 1,719 (7.5) | 1,698 (14.9) | 609 (6.1) |
| **Colorectal** | 2,601 (11.5) | 1,316 (11.4) | 832 (12.0) | 190 (11.1) | 190 (11.2) | 73 (11.9) |
| **Breast** | 1,610 (7.2) | 1,024 (8.8) | 292 (4.2) | 90 (5.2) | 154 (9.1) | 50 (8.2) |
| **Lung** | 4,021 (17.8) | 1,792 (15.5) | 1,683 (24.4) | 287 (16.7) | 199 (11.7) | 60 (9.8) |
| **Prostate** | 2,144 (9.5) | 1,154 (9.9) | 659 (9.5) | 152 (8.8) | 114 (6.7) | 65 (10.7) |
| **Other non-hepatic malignancy** | 13,268 (58.9) | 6,862 (59.2) | 3,837 (55.5) | 1,065 (61.9) | 1,120 (65.9) | 384 (63.0) |

Supplementary Table 4. 1, 5, and 10-year CIF estimates (%) by first decompensation event in cohort with decompensation (n=50,394).

|  |  | **Liver-related** | **Non-hepatic malignancy** | **CVD-related** | **Accident/ self-harm/ suicide/ homicide** | **Other** | **Liver transplant** |
| --- | --- | --- | --- | --- | --- | --- | --- |
| **Decompensation Type** | **Year** | **CIF (%)** | **CIF (%)** | **CIF (%)** | **CIF (%)** | **CIF (%)** | **CIF (%)** |
| **Ascites** | 1 | 17.6 | 4.9 | 3.8 | 0.5 | 7.7 | 0.9 |
|  | 5 | 32.9 | 7.8 | 7.4 | 1.2 | 13.9 | 3.0 |
|  | 10 | 40.7 | 9.5 | 9.1 | 1.6 | 17.7 | 3.9 |
| **Hepatic encephalopathy** | 1 | 22.9 | 3.9 | 2.9 | 0.8 | 9.6 | 2.5 |
|  | 5 | 38.2 | 5.6 | 5.2 | 1.4 | 15.6 | 5.3 |
|  | 10 | 45.6 | 6.6 | 6.6 | 1.8 | 18.9 | 6.4 |
| **Hepatorenal syndrome** | 1 | 40.9 | 3.9 | 2.9 | 0.4 | 12.9 | 0.6 |
|  | 5 | 55.1 | 4.7 | 3.8 | 0.6 | 16.0 | 1.5 |
|  | 10 | 62.9 | 5.0 | 4.3 | 0.7 | 17.7 | 1.8 |
| **Jaundice** | 1 | 12.9 | 7.5 | 2.0 | 0.3 | 5.6 | 1.5 |
|  | 5 | 23.4 | 11.8 | 4.2 | 1.1 | 10.5 | 2.8 |
|  | 10 | 28.7 | 14.0 | 6.5 | 1.4 | 14.8 | 3.4 |
| **Variceal bleeding** | 1 | 5.9 | 0.8 | 0.7 | 0.1 | 1.6 | 0.8 |
|  | 5 | 19.6 | 2.4 | 2.6 | 0.6 | 6.9 | 5.0 |
|  | 10 | 31.6 | 4.0 | 4.2 | 1.1 | 10.9 | 8.2 |

**Supplementary Figure Legends**

Figure S1. Inclusion and exclusion criteria for creation of cohort. AI: Autoimmune. ALD: alcohol-associated liver disease. HBV: hepatitis B virus. HCV: hepatitis C virus. NAFLD: non-alcoholic fatty liver disease.

Figure S2. Cumulative incidence functions for patients with non-alcoholic fatty liver disease stratified by sex for the main causes of death and liver transplant as a competing risk. CVD: cardiovascular disease.

Figure S3. Cumulative incidence functions for patients with alcohol-associated liver disease stratified by sex for the main causes of death and liver transplant as a competing risk. CVD: cardiovascular disease.

Figure S4. Cumulative incidence functions for patients with hepatitis C virus stratified by sex for the main causes of death and liver transplant as a competing risk. CVD: cardiovascular disease.

Figure S5. Cumulative incidence functions for patients with hepatitis B virus liver disease stratified by sex for the main causes of death and liver transplant as a competing risk. CVD: cardiovascular disease.

Figure S6. Cumulative incidence functions for patients with autoimmune/other liver disease stratified by sex for the main causes of death and liver transplant as a competing risk. CVD: cardiovascular disease.


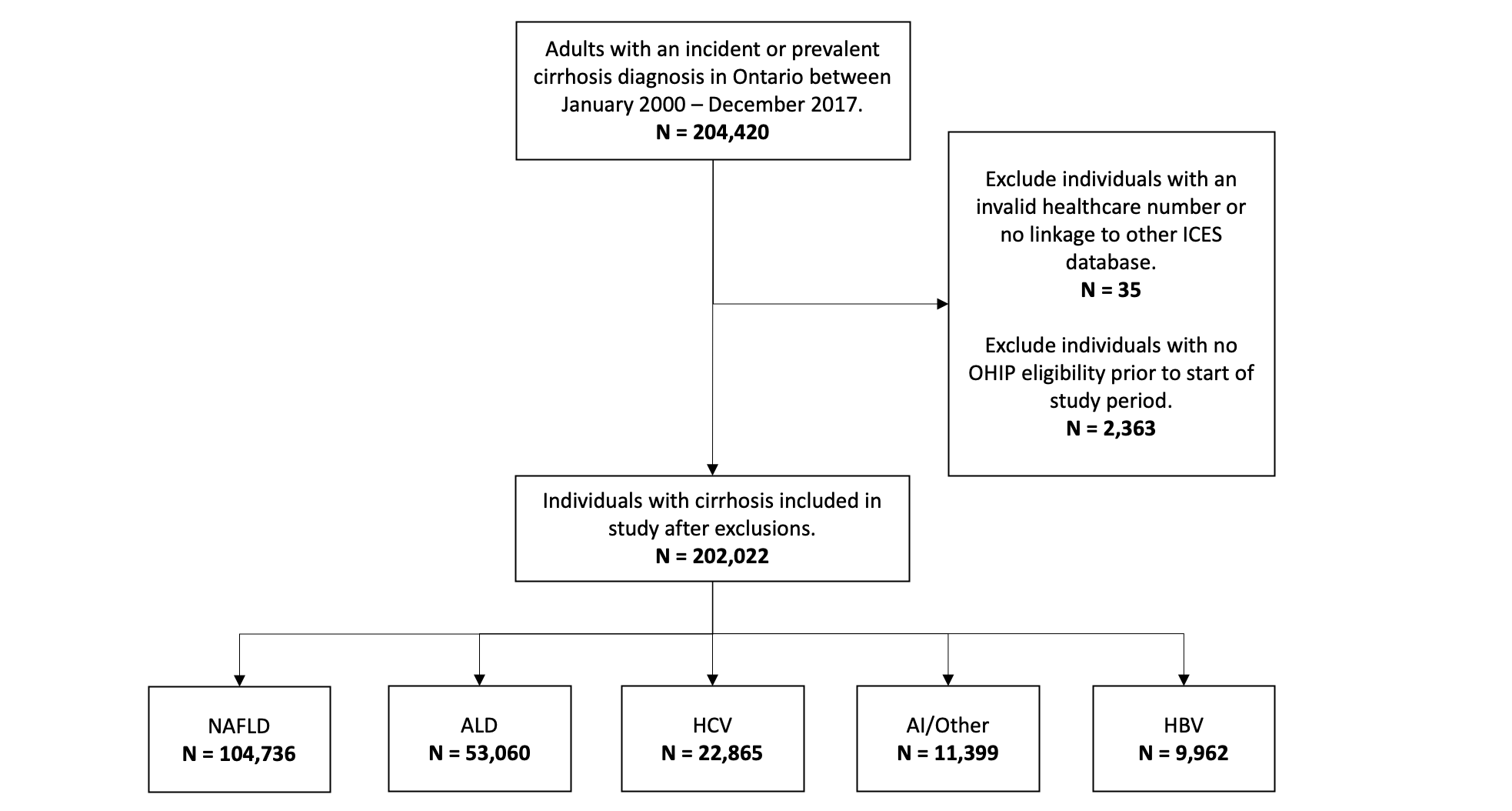


Figure S1


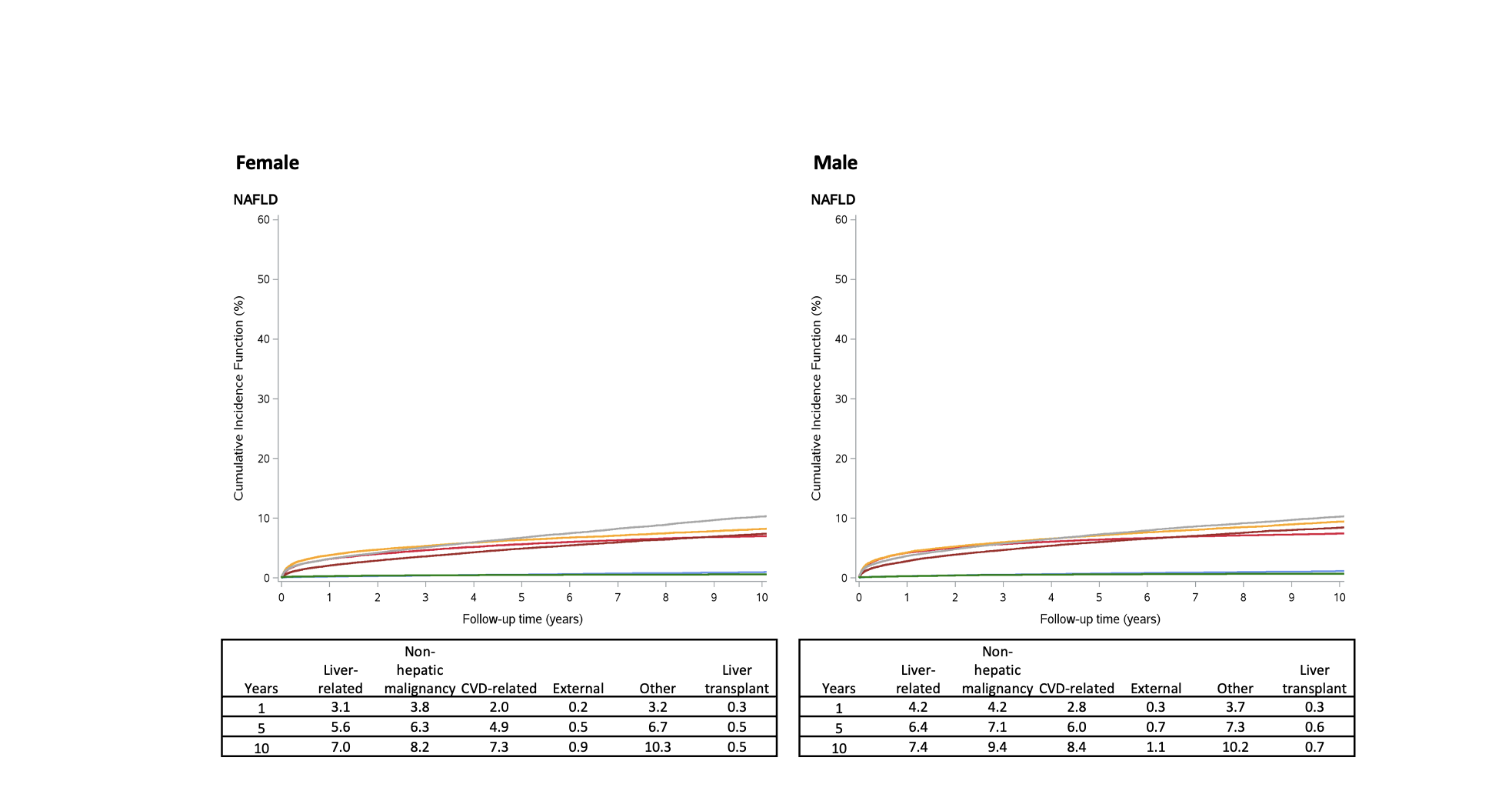


Figure S2


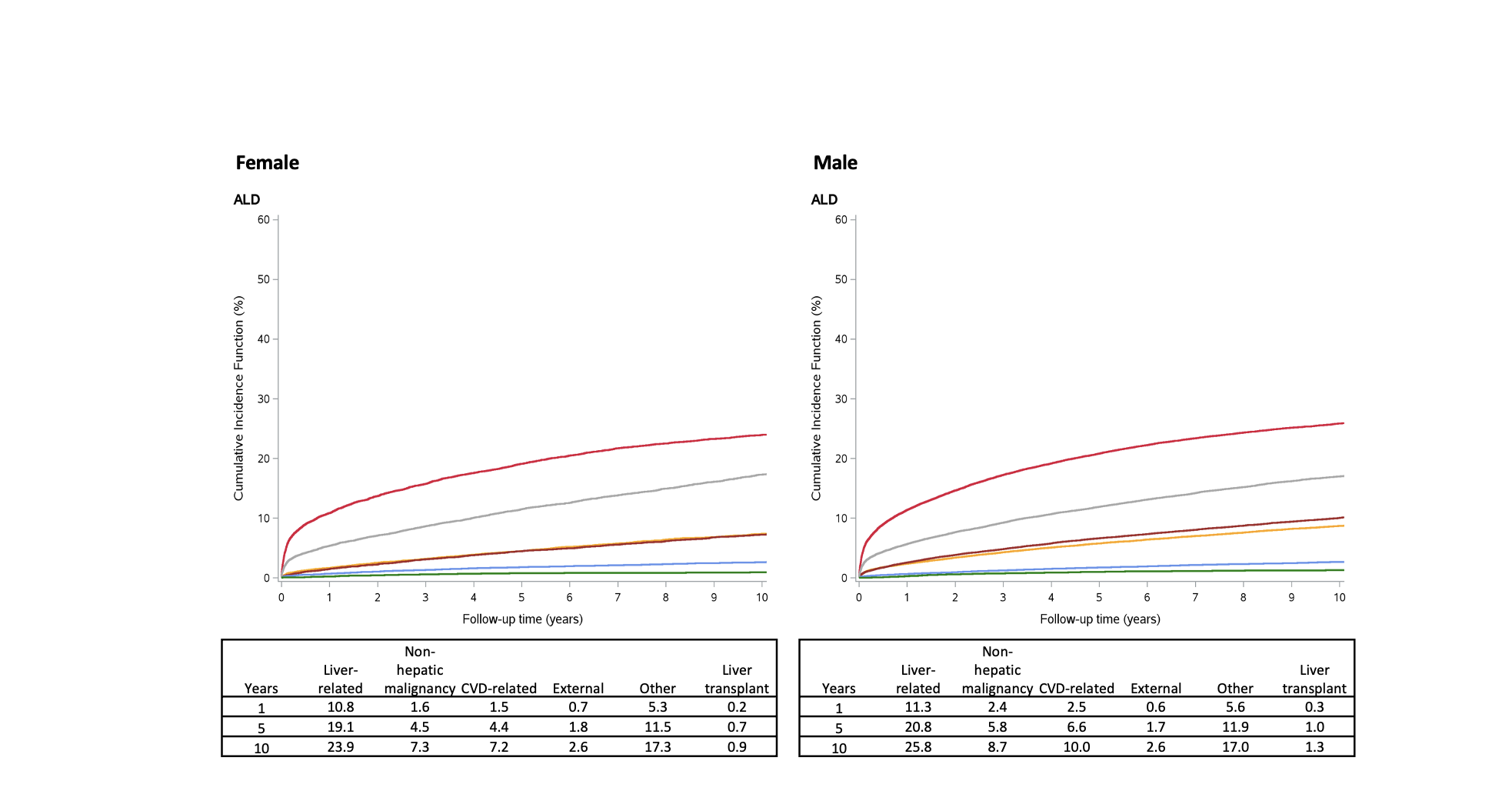


Figure S3


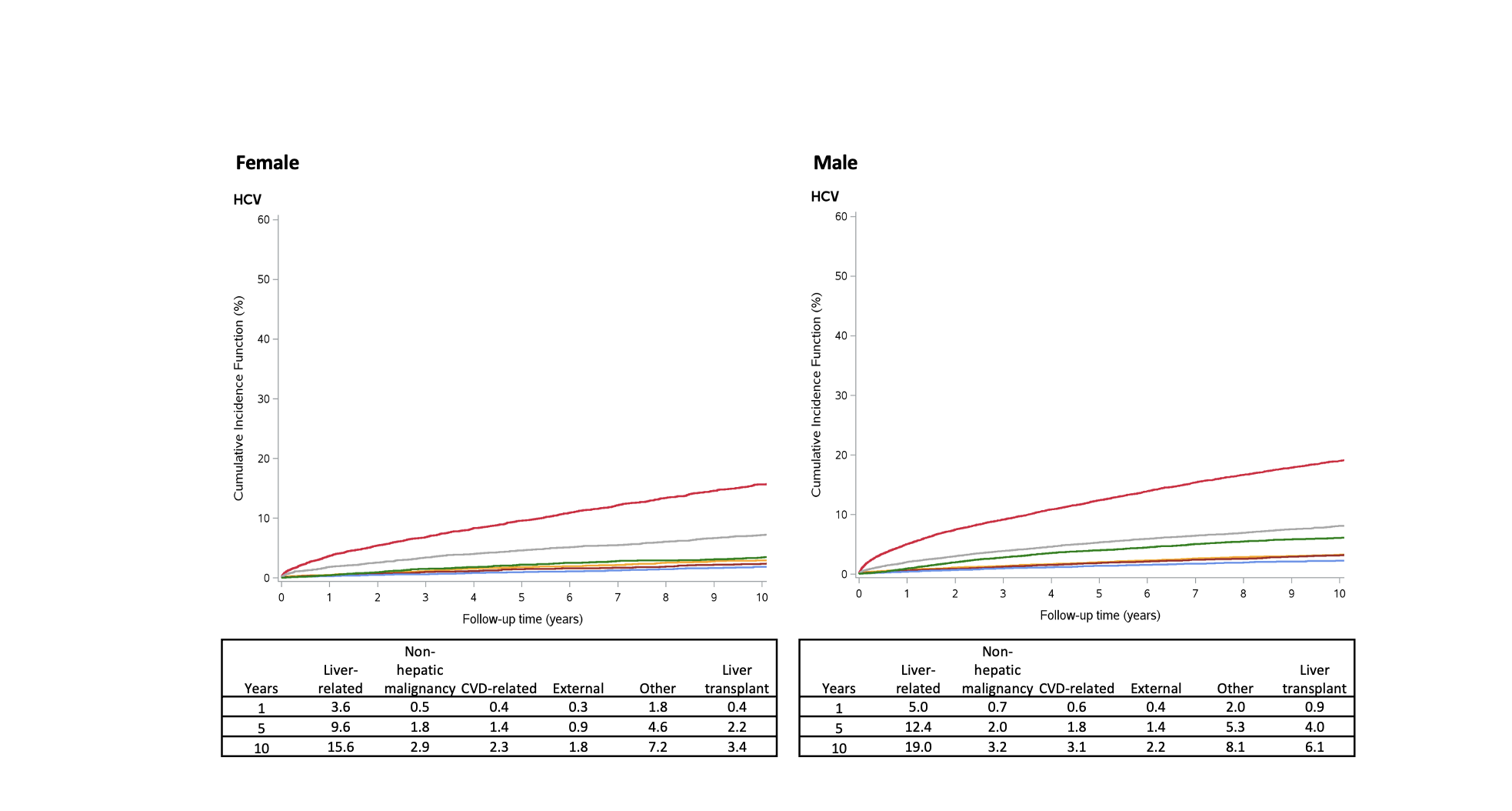


Figure S4


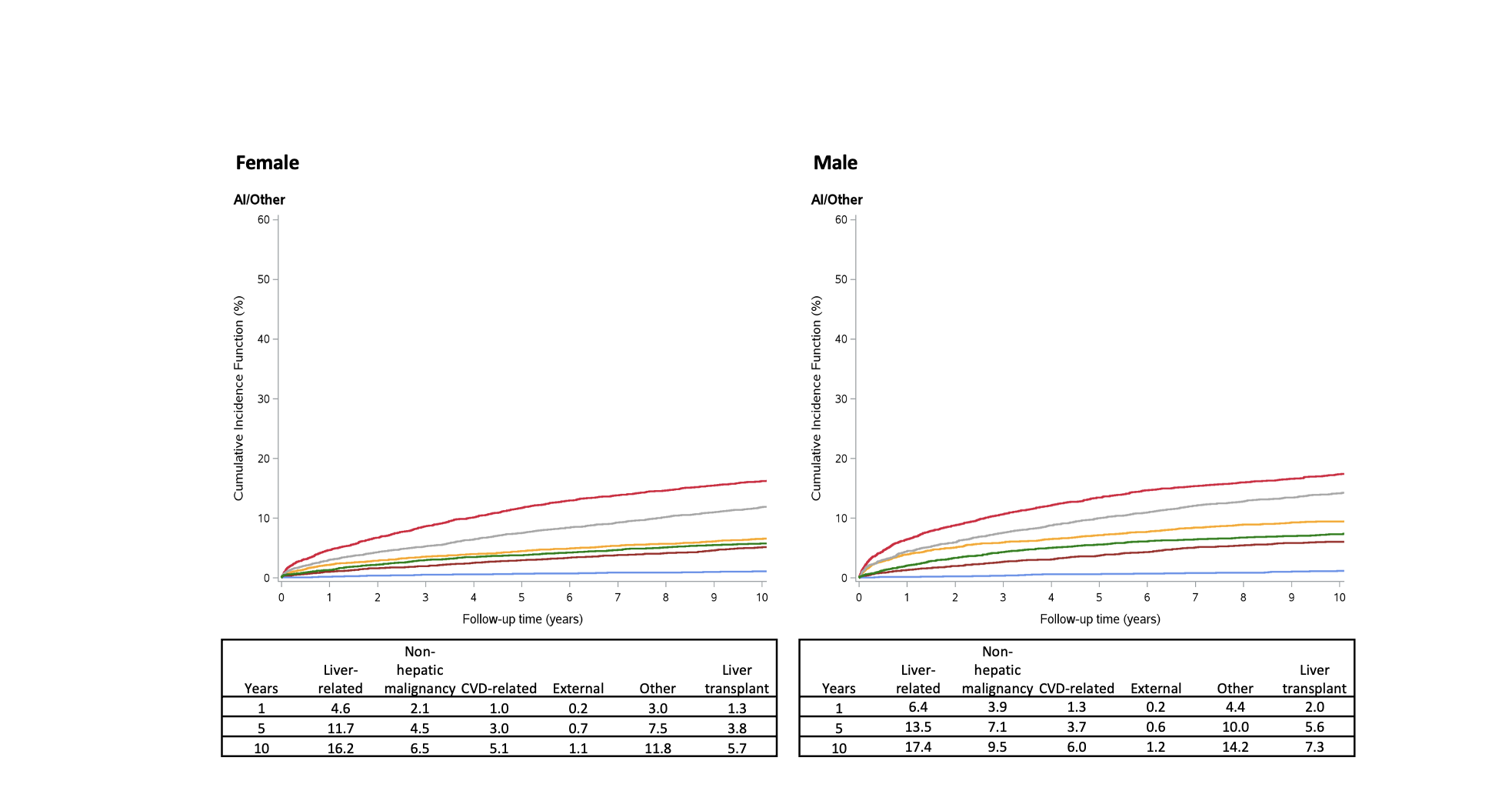


Figure S5


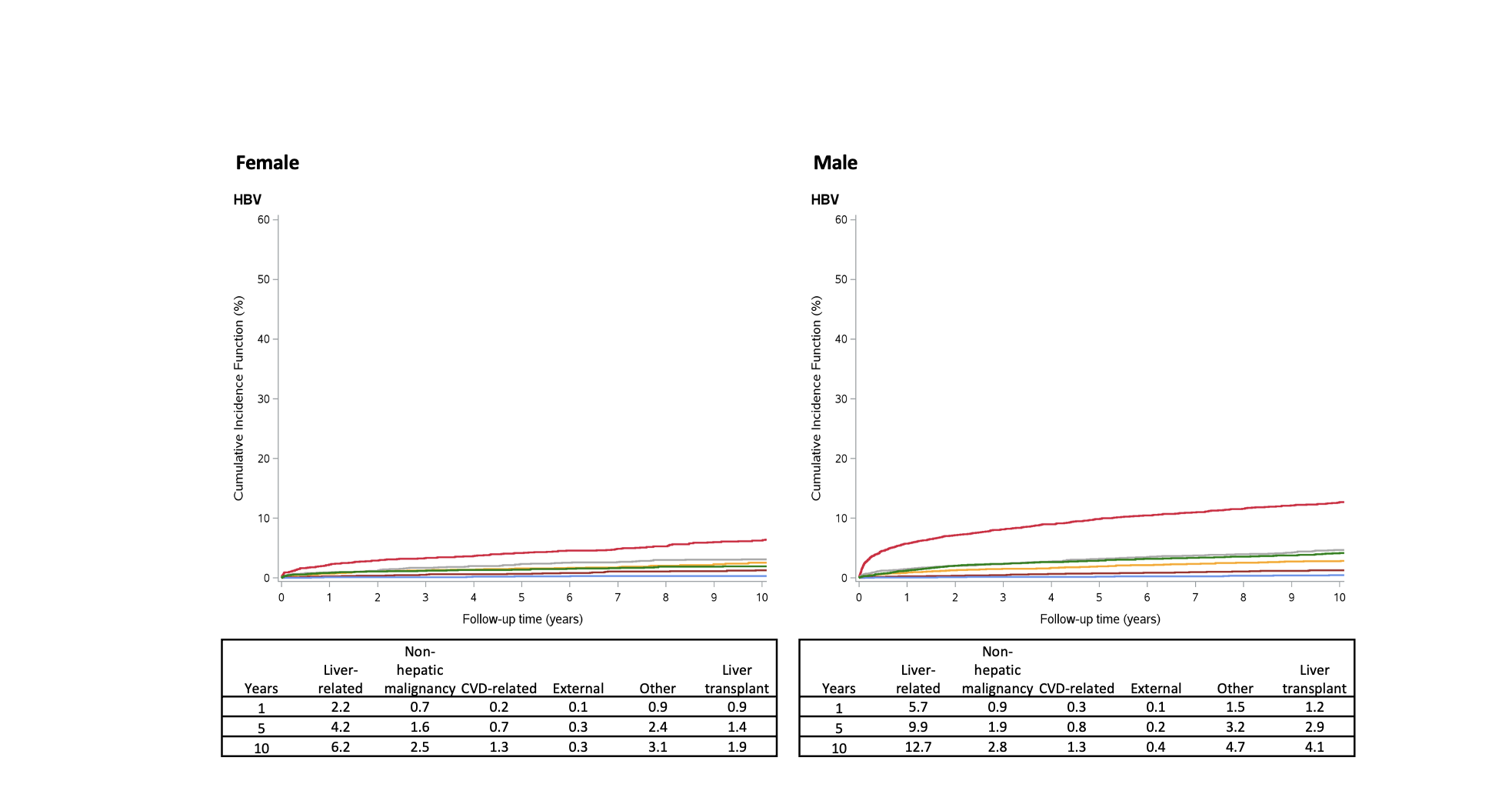
Figure S6
